# Compensatory strategies of dysphagia after anterior cervical spinal surgery: A case report

**DOI:** 10.1097/MD.0000000000039016

**Published:** 2024-07-19

**Authors:** Sung Joon Chung, Jun Ho Lee, Yunsoo Soh

**Affiliations:** a Department of Physical Medicine and Rehabilitation, Kyung Hee University Hospital at Gangdong, Seoul, Republic of Korea; b Department of Neurosurgery, Kyung Hee University Hospital, Seoul, Republic of Korea; c Department of Physical Medicine and Rehabilitation, Kyung Hee University Hospital, Seoul, Republic of Korea.

**Keywords:** anterior cervical discectomy and fusion, deglutition disorders, dysphagia, videofluoroscopic swallowing study

## Abstract

**Rationale:**

Dysphagia after anterior cervical discectomy and fusion (ACDF) is a common postoperative complication. However, information regarding rehabilitation strategies for postoperative dysphagia is limited. Herein, we report a compensatory strategy for treating dysphagia after ACDF.

**Patient concerns:**

A 65-year-old Asian male presented with left arm pain and weakness for more than 1 month. Magnetic resonance imaging of the cervical spine revealed degenerative disc lesions and spinal stenosis at the C3 to C7 levels. The patient underwent ACDF at the C3 to C5 levels and artificial disc replacement at the C5 to C7 levels by right side approach. After surgery, the patient complained of difficulty swallowing. A video fluoroscopic swallowing study (VFSS) detected swallowing dysfunction in the pharyngeal phase, revealing an asymmetric pharyngeal residue in the anterior–posterior view.

**Diagnosis:**

The patient was diagnosed with dysphagia after ACDF.

**Interventions:**

Based on the VFSS findings, the patient underwent swallowing rehabilitation therapy and compensatory techniques, such as head rotation to the weak right side and head tilting to the robust left side.

**Outcomes:**

After 2 months of rehabilitation with compensatory techniques, food moved smoothly towards the robust side, and the subjective symptoms of dysphagia improved.

**Lessons:**

Consequently, swallowing function post-ACDF surgery must be assessed; if unilateral dysphagia is detected, compensatory techniques may prove beneficial. This case study showed that, based on the objective findings of the VFSS, an effective swallowing compensation strategy can be established and applied to patients with postoperative dysphagia.

## 1. Introduction

Anterior cervical discectomy and fusion (ACDF) is a widely performed surgical procedure for treating cervical disease.^[1,2]^ Cervical disc herniation is a degenerative disease of the cervical spine in which a protruded disc compresses the exiting nerve and incurs segmental instability. ACDF via an anterior approach to the neck would be the most commonly implemented surgical option for this cervical spinal condition. In addition to common postoperative complications such as bleeding, infection, and pseudoarthrosis after ACDF, damage such as laryngeal nerve, muscle, soft tissue damage, and dysphagia may result from the anterior approach.^[3]^ Dysphagia is a disabling condition that may have a detrimental effect on quality of life. The incidence of dysphagia varies among studies owing to differences in the surgical method, individual patient characteristics, evaluation method, and follow-up period. In a previous report, the percentage of patients who experienced difficulty in swallowing immediately after ACDF surgery was relatively high.^[4]^ The rate of dysphagia reported after surgery ranges from 1% to 79%, but in most cases, these symptoms are in the short term and tend to improve naturally over time.^[4]^ Despite the limited information available on rehabilitative strategies for treating dysphagia following ACDF, this case report aims to evaluate and recommend an effective rehabilitation strategy for patients experiencing post-ACDF dysphagia.

## 2. Case presentation

**Figure 1.**
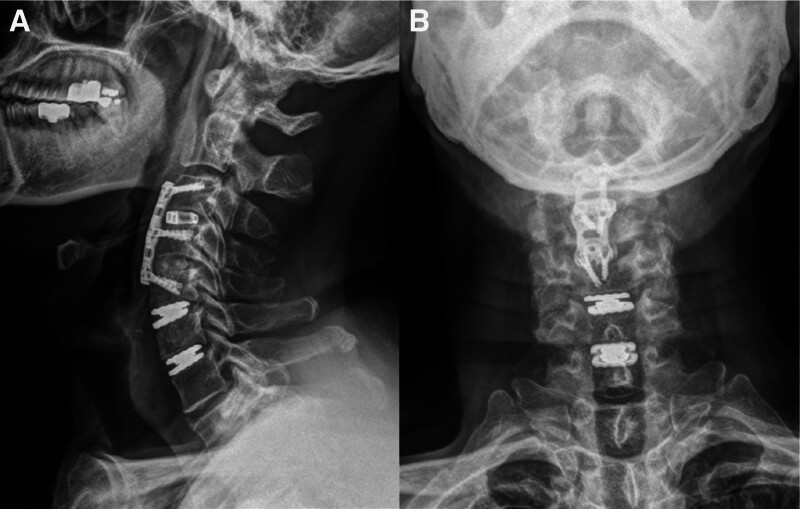
Postoperative cervical spine X-ray image. (A) Lateral and (B) Anterior–posterior projection showing anterior cervical fusion at the C3–C5 level and artificial disc replacement at the C5–C7 level.

**Figure 2.**
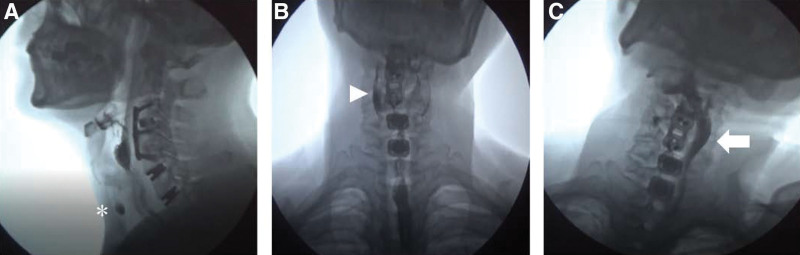
Video fluoroscopic swallowing study. (A) Lateral view. A large amount of pharyngeal residue is present in the pyriform sinus, and there is evidence of semi-solid bolus aspiration in the airway (asterisk). (B) Anterior–posterior view. A relatively large amount of pharyngeal residue is detected on the right side, compared with that on the left side (arrow head). (C) Anterior–posterior view with compensatory techniques. An intact passage of a food bolus is observed on the robust left side (arrow).

A 65-year-old Asian male patient (weight, 66 kg; height, 160 cm) presented with radiating pain in the left arm and weakness in the left upper extremity for 1 month. A manual muscle test of the shoulder and elbow revealed a grade of 2. Neurological examinations for myelopathy, such as Hoffman sign, Lhermitte sign, biceps, knee jerk, and ankle clonus, showed normal findings. No relevant medical history and no past surgical or injection interventions were found. The patient underwent cervical magnetic resonance imaging, which revealed multiple spinal stenoses and degenerative disc herniation at the C3 to C6 level. The patient underwent ACDF at the C3 to C5 levels and artificial disc replacement at the C5 to C7 levels by right side approach (Fig. 1). The day after surgery, the patient complained of difficulty swallowing, specifically difficulty in passing food into the esophagus when swallowing solids and foreign-body sensations. A video fluoroscopic swallowing study (VFSS) detected swallowing dysfunction in the pharyngeal phase (Fig. 2A). This study revealed large amounts (>50%) of pharyngeal residue, reduced laryngeal elevation, reduced upper esophageal sphincter opening, and evidence of aspiration during swallowing of semisolids. Right-sided asymmetric vallecular and pyriform sinus residues were detected in the anterior–posterior view of the VFSS, with a relatively preserved passage of the food bolus on the left side, compared with that of the right side (Fig. 2B). Based on the VFSS findings, the patient underwent swallowing rehabilitation therapy and was educated on compensatory techniques, such as head rotation to the right side and head tilting to the left side, for unilateral laryngeal weakness. After 2 months of rehabilitation with compensatory techniques, food moved smoothly towards the robust side and improved the subjective symptoms of dysphagia (Fig. 2C). A VFSS follow-up study was conducted 6 months after surgery, and laryngeal elevation improved, and no aspiration or penetration was observed. However, when swallowed without the compensatory technique, a large amount of residue, similar to prior observations, was noted on the right side. This residue was only removed after applying the compensatory technique and multiple swallowing attempts.

## 3. Discussion

Dysphagia is a common complication of ACDF. In most patients who undergo ACDF, swallowing difficulties fully recover within a few days postoperatively. However, these difficulties can persist for weeks, months, or years. A previous study describes that dysphagia, with a mean incidence of 19.4%, leads to adverse effects such as decreased quality of life and aspiration pneumonia.^[4]^ Dysphagia, as a postoperative complication of ACDF, is multifactorial and multivariable. Previous studies have reported that dysphagia after ACDF occurs when the cervical level is higher as multiple segments undergo operation and when cervical soft tissue edema is severe, even 48 hours after surgery, which causes dysphagia.^[5]^ Other factors include patient sex, surgical technique, variety of ACDF devices implemented, type of graft utilized, application of recombinant human bone morphogenetic protein-2 (rhBMP-2), and administration of corticosteroids.^[6]^ These factors can lead to recurrent laryngeal nerve injury, local muscle or soft tissue damage during surgery, and postoperative edema, which causes unilateral paralytic dysphagia after ACDF.

Postoperative dysphagia after anterior cervical surgery predominantly manifests during the pharyngeal phase of swallowing. Swallowing dysfunction presents as an impairment or significant delay in initiating pharyngeal swallowing, aspiration of ingested material, nasopharyngeal regurgitation, and residual estate within the pharyngeal cavity after deglutition. Each category intrinsically pertains to the pharyngeal phase of the swallowing mechanism. Dysphagia rehabilitation after ACDF has been introduced and includes diet modification, voluntary control of swallowing, and laryngeal elevation exercises to improve the range of oral or pharyngeal structural movement. However, no reports have detailed compensatory techniques.

Our patient underwent VFSS using the head rotation and tilt compensation technique, and the food bolus was confirmed to move to the robust side in the anterior–posterior view. The following mechanism explains this phenomenon: Head rotation is a compensatory strategy for patients with unilateral pharyngeal and laryngeal weakness.^[7]^ Clinicians may recommend that patients turn their heads laterally, mimicking the gesture of looking over the shoulder. When directed towards the side of impairment, this maneuver efficaciously reroutes the bolus to the contralateral pharyngeal side, which is more robust. Furthermore, head rotation has been identified as an advantageous technique to diminish upper esophageal sphincter pressure on the side opposite the direction of the head turn, thereby facilitating an augmented extension and duration of upper esophageal sphincter opening. In patients with compromised laryngeal closure, head rotation constricts the laryngeal entrance and enhances vocal-fold closure by exerting extrinsic pressure. The head tilt maneuver is implemented in patients experiencing unilateral pharyngeal and oral weakness.^[8]^ Clinicians advise such patients to execute movements characterized by tilting the head laterally, simulating an attempt to bring the ear close to the shoulder. This technique is advantageous, as it efficiently facilitates redirection of the bolus towards the more robust side of the pharyngeal cavity. Consequently, swallowing function post-ACDF surgery must be assessed, and accurate diagnosis of patients’ weak sides through the anteroposterior view of the VFSS is essential. If unilateral dysphagia is detected, compensatory techniques may be beneficial. Unlike general dysphagia rehabilitation, compensatory techniques allow faster and safer eating, minimize the risk of aspiration, and do not require adjustments to the dysphagia diet. This case study showed that based on the objective findings of the VFSS, an effective swallowing rehabilitation strategy can be established and applied to patients with postoperative dysphagia.

## 4. Conclusion

This is the first study to report that the compensation technique improves the unilateral symptoms of pharyngeal phase dysphagia in patients who underwent ACDF surgery. This report suggests that in patients with unilateral dysphagia after ACDF, not only dysphagia rehabilitation but also compensation techniques provide the possibility for patients to eat more safely. Further research is necessary to develop more detailed and specific rehabilitation strategies for unilateral dysphagia, beyond compensatory techniques.

## Acknowledgments

We thank the patient for providing this information.

## Author contributions

**Visualization:** Sung Joon Chung.

**Supervision:** Yunsoo Soh.

**Writing – original draft:** Yunsoo Soh.

**Writing – review & editing:** Yunsoo Soh.

**Investigation:** Jun Ho Lee.
